# Long-Term Functional Outcomes After Prehabilitation in Frail Older Adults Undergoing Colorectal Cancer Surgery: A One-Year Prospective Cohort Study

**DOI:** 10.3390/jcm15124731

**Published:** 2026-06-18

**Authors:** Małgorzata Dobrzycka, Patryk Wołoszyn, Magdalena Prud, Ksawery Bieniaszewski, Piotr Spychalski, Katarzyna Gierat-Haponiuk, Jarosław Kobiela

**Affiliations:** 1 Department of Surgical Oncology, Transplant Surgery and General Surgery, Medical University of Gdańsk, 80-210 Gdańsk, Poland; 2 Department of Clinical Physiotherapy, Medical University of Gdańsk, 80-210 Gdańsk, Poland

**Keywords:** frailty syndrome, preoperative rehabilitation, geriatric oncology, functional recovery, colorectal surgery

## Abstract

**Background**: Frailty is associated with adverse postoperative outcomes and functional decline in older adults undergoing colorectal cancer (CRC) surgery. The long-term course of frailty and functional outcomes among patients undergoing prehabilitation before CRC surgery remains insufficiently investigated. **Methods**: This prospective observational cohort study evaluated long-term functional and physiological outcomes in older adults with frailty syndrome undergoing colorectal cancer (CRC) surgery who participated in a structured prehabilitation program. Forty-one patients aged >70 years were assessed before prehabilitation and at one-year follow-up. Frailty (the Clinical Frailty Scale [CFS] and the 5-item Frailty Index [5-FI]), physical activity, postural function, respiratory parameters, and functional performance (the 6 min walk test [6MWT] and the Timed Up and Go [TUG] test) were evaluated. **Results**: Of the 93 eligible patients, 41 completed the one-year follow-up and were therefore included in the final analysis. A small but statistically significant increase in frailty was observed using 5-FI (mean difference = 0.029, *p* = 0.012), with no significant change in CFS. Postural function improved (*p* = 0.031), while physical activity and functional performance remained stable (6MWT: 392.71 vs. 384.36 m, *p* = 0.885; TUG: 12.36 vs. 10.42 s, *p* = 0.051). A significant reduction in pre- and post-exercise oxygen saturation was observed; however, the magnitude of change (before: −1.25%, *p* = 0.006; after: −0.91%, *p* < 0.001) was small and of uncertain relevance. **Conclusions**: Over a one-year follow-up of prehabilitated CRC patients with frailty, their functional performance remained stable despite a subtle progression of frailty. These findings suggest a dissociation between physiological vulnerability and functional status. Due to the observational design of the study and the lack of a control group, the results should be interpreted as descriptive rather than causal.

## 1. Introduction

Colorectal cancer (CRC) remains one of the most common malignancies worldwide, with a particularly high incidence in patients over 70 years of age [1]. Advances in surgical techniques (minimally invasive, robotic surgery) and oncologic therapies have improved survival rates, yet a significant proportion of patients experience long-term impairments in physical function, cardiorespiratory fitness, and quality of life following treatment [2,3].

Frailty, a multidimensional syndrome characterized by reduced physiological reserve and increased vulnerability to stressors such as a surgery, affects approximately 25–50% of older adults undergoing colorectal cancer surgery [4,5]. It has emerged as a critical determinant of both short- and long-term postoperative outcomes, including a higher incidence of postoperative adverse events, delayed recovery, and diminished functional capacity [5,6,7]. Frailty has also been strongly associated with increased mortality in colorectal cancer patients, with Normann et al. reporting significantly higher 90-day mortality (OR: 4.97; 95% CI: 1.06–23.28; *p* = 0.042) and one-year mortality (OR: 4.39; 95% CI: 1.86–10.34; *p* = 0.0007) among frail individuals [4]. The prevalence of frailty rises substantially with age, and it is especially relevant in older adults undergoing major oncological surgery [5,8,9]. In addition to short-term postoperative outcomes, there is a growing need to understand long-term physiological and functional trajectories in frail older adults undergoing CRC surgery. Previous studies have predominantly focused on perioperative morbidity, whereas data on sustained functional independence, respiratory mechanics, and exercise tolerance remain limited beyond the early postoperative period [10]. Therefore, evaluating the durability of prehabilitation-associated benefits in geriatric oncology populations is essential for optimizing recovery pathways and tailoring postoperative supportive care strategies.

In recent years, prehabilitation has emerged as a proactive approach aimed at optimizing patients’ functional capacity prior to surgery. Multimodal prehabilitation programs typically combine structured physical exercise, respiratory training, nutritional optimization, and psychological support to enhance physiological reserve before exposure to surgical stress. Several randomized and observational studies have demonstrated that prehabilitation may improve early postoperative outcomes, including reduced complications, shorter hospital stays, and faster recovery of functional mobility [11,12,13].

Despite the growing body of evidence supporting short-term benefits of prehabilitation, knowledge regarding the durability of these effects in older prehabilitated adults remains limited. Most available studies evaluate outcomes within 30–90 days after surgery, focusing primarily on perioperative morbidity and early functional recovery [14,15]. However, the long-term trajectory of functional performance in frail older adults following CRC surgery remains insufficiently investigated [16]. Understanding whether functional gains achieved through prehabilitation persist beyond the immediate postoperative period is essential for assessing the true clinical value of these interventions.

Furthermore, frailty is a dynamic process that may evolve over time due to aging, cancer progression, and treatment-related factors. Long-term follow-up studies are therefore necessary to distinguish between physiological decline related to natural aging and functional deterioration potentially preventable through targeted preoperative conditioning.

Based on these considerations, we hypothesized that functional performance in frail older adults undergoing colorectal cancer surgery following prehabilitation would remain relatively preserved over a one-year follow-up period, despite potential progression of physiological frailty.

This prospective observational cohort study aimed to investigate longitudinal changes in functional and physiological outcomes in older adults with frailty undergoing CRC surgery following a structured prehabilitation program. Specifically, we investigated changes in frailty status, functional performance, physical activity, and cardiopulmonary parameters over a 12-month follow-up period.

## 2. Materials and Methods

### 2.1. Study Design and Participants

This study was designed as a prospective, observational cohort study evaluating longitudinal functional and physiological trajectories of outcomes in older adults with frailty syndrome undergoing CRC surgery who participated in a structured prehabilitation program prior to surgery.

The study was conducted at single tertiary Colorectal Cancer Unit (the University Clinical Centre in Gdańsk, Poland). Patients aged over 70 years who underwent curative surgical treatment for CRC were enrolled consecutively between 1 January and 31 December 2023. Participants were followed for 12 months after completion of treatment.

Primary outcomes were changes in frailty status assessed by the Clinical Frailty Scale (CFS) and 5-item Frailty Index (5-FI) in patients who received prehabilitation before CRC surgery.

Secondary outcomes included physical activity level, postural function, respiratory parameters, and functional performance measured using the 6 min walk test (6MWT) and Timed Up and Go (TUG) test, as well as exercise-related cardiopulmonary responses.

The study was conducted in accordance with the Declaration of Helsinki and approved by the Institutional Review Board of the Medical University of Gdańsk, Poland (approval number: KB/7/2024, 19 January 2024). All participants provided written informed consent prior to inclusion in the study.

**Figure 1:**
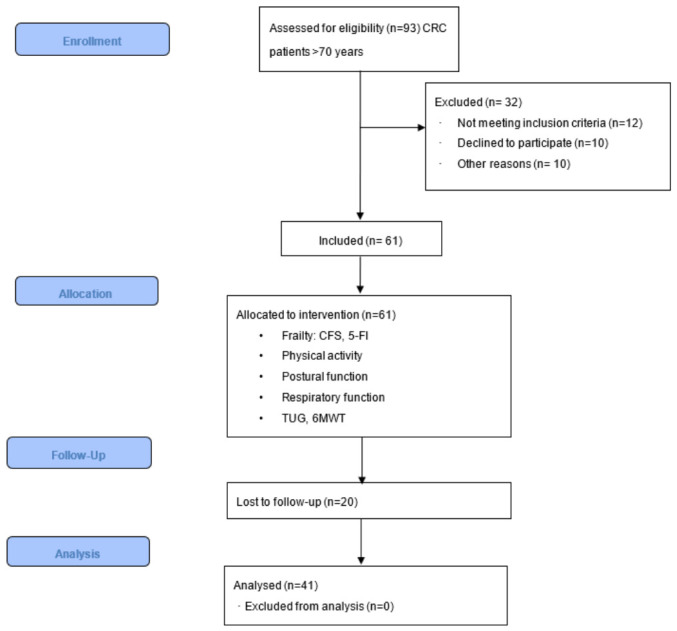
Study design and patient flow.

The overall study design and patient flow are presented in Figure 1 (Study design and patient flow).

### 2.2. Inclusion/Exclusion Criteria

Eligible participants were patients aged ≥70 years diagnosed with stage I–III CRC (locally advanced CRC) and scheduled for elective surgical treatment. Tumor staging was determined according to the TNM classification system based on preoperative imaging and histopathological evaluation. Inclusion criteria were age >70 years with histologically confirmed CRC (stage I–III), diagnosis of frailty syndrome, eligibility for elective surgical treatment, and ability to participate in prehabilitation training. Exclusion criteria included palliative treatment, metastatic disease (CRC stage IV), severe cognitive impairment preventing cooperation, contraindications to physical exercise, inability to complete follow-up assessment, or incomplete data.

A total of 41 patients completed the full prehabilitation program and the one-year follow-up assessment and were included in the final analysis.

### 2.3. Data Collection

Changes in frailty and physiological parameters were assessed over a one-year follow-up period after treatment for CRC. To assess frailty, we used CFS and 5-FI [17,18]. CFS is a well-established tool for the assessment of frailty on a 9-point scale, assessing frailty based on functional independence, comorbidities, and physical fitness, with the severity of frailty increasing with each number [18]. 5-FI is based on the presence of five criteria: chronic obstructive pulmonary disease (COPD) or recent pneumonia, congestive heart failure, diabetes mellitus, hypertension requiring medication, and non-independent functional status. Non-independent functional status was defined as requiring assistance in at least one activity of daily living [19].

Physical and physiological functions were recorded using physical activity scores postural function scores; and chest circumference in inspiration, expiration, and its delta. The Clinical Physical Activity Scale was used to evaluate physical activity. The questionnaire measures walking, moderate, and vigorous physical activity during the previous week, and results are expressed in numbers according to standardized scoring procedures. Physiological parameters were assessed during the 6MWT (measurements of heart rate (HR), systolic blood pressure (SBP), diastolic blood pressure (DBP), mean arterial pressure (MAP), SpO_2_ before and after 6MWT, and Borg scale at rest and post-exercise). Peripheral oxygen saturation (SpO_2_) was measured using a pulse oximeter before and immediately after completion of the 6MWT.

Functional mobility was assessed using two standardized tests: the TUG test and 6MWT. The TUG test was used to evaluate functional mobility and balance [20]. Patients were instructed to stand up from a chair, walk three meters, turn around, walk back, and sit down. The time required to complete the task was recorded in seconds. Exercise capacity was assessed using the 6MWT, conducted according to standardized guidelines [21,22]. The total walking distance achieved in six minutes was recorded in meters.

Postural function was assessed using a standardized physiotherapeutic evaluation including trunk alignment, balance control, and postural stability during standing and gait tasks. The assessment was based on a semi-quantitative scoring system that ranged from 0 to 2 points, with higher values indicating better postural performance. All assessments were performed by trained physiotherapists using consistent protocols.

The multimodal prehabilitation protocol was based on established prehabilitation protocols and consisted of individualized low-to-moderate intensity aerobic training, resistance-based functional exercises, breathing exercises targeting thoracic mobility, and balance-oriented neuromuscular training, implemented at least 2 weeks before the surgery [10,14,23]. Participants were instructed by a physiotherapist and performed home-based components adapted to patient-reported fatigue and comorbidity burden in daily sessions of 30–60 min on average. Nutritional counselling and education on daily physical activity were also incorporated to improve baseline physiological reserve prior to surgery [24]. Each patient participated in an individualized physiotherapy program tailored to their baseline functional status, physical capacity, and specific rehabilitation needs according to initial tests. During the initial session, the physiotherapist experienced in geriatrics and oncology assessed baseline functional parameters, including frailty status, physical performance, and cardiorespiratory function. Patients performed moderate-intensity aerobic exercise, including treadmill walking or cycling. Exercise intensity was adjusted individually according to baseline functional capacity and tolerance. Sessions typically lasted 20–30 min and were conducted at 50–70% of estimated maximal HR. Resistance exercises targeting major muscle groups were performed using body weight or light resistance equipment. Inspiratory muscle training and breathing exercises were incorporated to improve pulmonary function and respiratory muscle strength. Neuromuscular exercises focusing on posture correction, trunk stability, and balance control were implemented to improve biomechanical efficiency and reduce fall risk [21,25]. Throughout the prehabilitation period, the physiotherapist met with the patient at regular intervals to monitor progress and adjust the exercise plan as needed. The same set of assessments was repeated at the end of the physiotherapy program and after a one-year follow-up.

### 2.4. Bias Control

Several strategies were implemented to minimize potential sources of bias. First, all functional assessments were performed using standardized protocols and conducted by trained physiotherapists. Second, the same assessment procedures were applied at baseline and follow-up evaluations. However, potential selection bias and survivorship bias cannot be fully excluded because only patients who completed the one-year follow-up assessment were included in the final analysis.

### 2.5. Sample Size Considerations

Due to the exploratory nature of the study and the limited number of patients who met the inclusion criteria during the study period, no formal a priori sample size calculation was performed. The sample size therefore represents a consecutive cohort of eligible patients who completed the prehabilitation program and one-year follow-up assessment.

### 2.6. Statistical Analysis

Continuous variables are presented as means ± standard deviations. After confirming the normal distribution of the data using the Shapiro–Wilk test, subgroup analysis was performed using paired t-tests to compare parameters before prehabilitation and after the one-year follow-up. A *p* < 0.05 was considered statistically significant. Potential confounding variables such as age, comorbidities, and surgical approach were considered during the interpretation of the results. SAS software (v.9.4; SAS Institute, Cary, NC, USA) was used for the analyses.

## 3. Results

### Patient Demographics

From January 2023 to December 2023, a total of 93 patients (aged over 70) underwent curative resection for colon or rectal cancer in the large Colorectal Cancer Unit, of which 41 met the inclusion criteria and agreed to the 1-year follow-up postsurgery and were included in the analysis. The remaining patients were excluded due to their not meeting the eligibility criteria, refusing to participate in the follow-up, or having incomplete data, which may have introduced potential selection and survivorship bias.

Among the included participants, 6 patients (14.6%) were classified as having favorable frailty status (5-FI = 0), 18 patients (43.9%) as moderate (5-FI = 1), and 17 patients (41.5%) as severe (5-FI ≥ 2). The mean age of the cohort was 75 years (median: 74 years), with 34.1% of the patients being female (Table 1).

No significant change was observed in CFS (mean diff = 0.0732 ± 0.6079, *p* = 0.445) whereas 5-FI increased slightly but significantly (mean diff = 0.0293 ± 0.0716, *p* = 0.012) in the one-year follow up.

Table 2 summarizes the physical and physiological functions at baseline before the prehabilitation and after the 1-year follow-up.

There is no deterioration in physical activity (mean diff = −0.27, ±1.1123, *p* = 0.380), with a significant improvement in postural function (mean diff = 0.40, ±0.9685, *p* = 0.031). Respiratory function remains unchanged over the year after the surgery.

Functional capacity analysis revealed no significant differences in 6MWT distance (392.71 m vs. 384.36: m, *p* = 0.885) or TUG test time (12.36 s vs. 10.42 s, *p* = 0.051).

A statistically significant decrease in SpO_2_ was observed both before and after the 6MWT. The magnitude of change was small (~1%) and remained within the normal physiological range.

A detailed summary of the functional capacity assessment is presented in Table 3.

## 4. Discussion

The prospective cohort study evaluated the long-term functional and physiological trajectories of older adults with frailty syndrome undergoing CRC surgery who participated in a structured prehabilitation program with a follow-up period of twelve months. Long-term data in this population remain scarce, and our findings contribute to the understanding of recovery trajectories beyond the immediate postoperative period [4,26,27].

A notable observation in our study was the discrepancy between frailty measures. This principal finding of the study indicates that in patients aged > 70 years old undergoing preoperative prehabilitation and surgery, frailty measured by the 5-item FI subtly worsens over the 12 months following CRC treatment but without a change in CFS. The divergence between 5-FI and CFS may suggest progression of subclinical physiological vulnerability without overt functional decline. The increase in 5-FI suggests accumulation of comorbid deficits, whereas stable CFS indicates preserved functional independence [17,28,29]. According to the results of a previous study, even a slight increase in 5-FI over 1 year is associated with increased mortality risk [7], whereas the unchanged CFS scores in our patients suggest stabilization in functional capacity despite the negative impact of surgery in frailty patients reported in the literature, indicating a possible good ability to mobilize and perform activities of daily living despite frailty [4,30,31,32]. Consequently, this pattern may represent an early stage of frailty progression, where physiological reserve declines without substantial deterioration in daily functional status. This finding suggests that while subclinical physiological vulnerability may progress over time, global functional independence can remain preserved in a proportion of patients. The CFS reflects overall functional status and daily activity capacity, whereas the 5-FI captures the accumulation of comorbidities and physiological deficits [17,19,28]. The divergence between these measures may therefore reflect the multifactorial nature of frailty and highlights the importance of using multiple assessment tools when evaluating frailty trajectories in geriatric oncology cohorts [6,7,33].

Secondly, we did not observe a statistically significant deterioration in physical activity scores (1.97 vs. 1.63, mean diff = −0.27 ± 1.1123, *p* = 0.380), with a modest but significant improvement in postural function scores (0.67 vs. 0.93, mean diff = 0.40 ± 0.9685, *p* = 0.031). These findings provide insight into the multimodal relationship between physiological frailty progression and the preservation of functional independence in older surgical patients [10,33,34]. Other recent studies also support reduced postoperative complications and improved short-term functional outcomes in high-risk CRC patients undergoing prehabilitation [14,35,36]. The observed better postural function despite similar physical activity, often relying on neuromuscular control, balance, and core stability, which are the elements of the prehabilitation program, may also be attributed to the positive effect of prehabilitation [37,38,39]. Importantly, the results should be interpreted cautiously because the study did not include a control group. Consequently, the findings should not be interpreted as evidence of a causal effect of prehabilitation but rather as observations of functional trajectories in patients who underwent surgery following a prehabilitation program. This distinction is particularly relevant in observational cohort studies, where multiple factors may influence postoperative recovery and long-term functional outcomes.

Interestingly, we observed the stability of overall functional mobility over the 12-month follow-up period, as reflected by stable 6MWT distance results and no difference in TUG times. In frail populations, functional performance typically declines following major surgery due to reduced physiological reserve, prolonged recovery, and postoperative complications [4,16,33]. Therefore, the maintenance of baseline mobility in this cohort may reflect a relatively favorable functional trajectory in comparison with the expected natural decline observed in similar cohorts [10]. However, given the observational design of the study, these findings should be interpreted as associations rather than direct effects of the prehabilitation intervention. In the literature, prehabilitation is related to increased 6MWT results in short-term observation, but the effectiveness of this strategy in long-term observation and for patients aged ≥70 years after surgery for CRC is unknown [14,40,41].

Another important observation was a significant improvement in postural function, which may have important clinical implications. Postural stability and trunk control are critical components of mobility and fall prevention in older adults [42,43]. Improvements in these parameters may reflect neuromuscular adaptations associated with targeted physiotherapy interventions, particularly those focusing on balance training and core stabilization [10,33]. Enhanced postural control may contribute to the preservation of mobility and independence during long-term recovery after major abdominal surgery [10].

A statistically significant reduction in SpO_2_ measured before and after the 6MWT was also observed during the follow-up period. The observed magnitude of change (~1%) falls within the expected physiological variability in pulse oximetry measurements. Therefore, this finding should be interpreted with caution, as it may not represent clinically meaningful deterioration in cardiopulmonary function in this cohort. Nevertheless, these results highlight the importance of monitoring respiratory function during long-term recovery in older surgical patients. Reduced cardiopulmonary efficiency during exertion has been previously reported in older cancer survivors and may influence long-term physical resilience [44,45]. Older adults undergoing oncologic surgery often experience progressive declines in pulmonary function due to factors such as aging, reduced physical activity, and the physiological impact of cancer and its treatment [46,47,48].

Importantly, the stability of functional capacity observed in this study contrasts with the natural trajectory of frailty-associated decline commonly described in geriatric cohorts [27]. Previous longitudinal studies have demonstrated that frail older adults often experience progressive reductions in mobility and exercise tolerance following major surgical interventions [49,50]. Our findings therefore raise the possibility that prehabilitation may play a role in mitigating long-term functional deterioration, although further controlled studies are required to confirm this hypothesis.

The findings of the present study should also be interpreted in the context of the high proportion of patients undergoing open surgical procedures at the time of conducting the study, which represented the majority of cases in this cohort. Open colorectal surgery is associated with greater surgical stress and longer recovery times compared with minimally invasive techniques [51,52]. This factor may have influenced the observed long-term functional trajectories and should be considered when interpreting the results.

The results of our study may be directly applicable in modern clinical practice. The ERAS protocol for patients aged ≥70 years may be additionally implemented with long-term postoperative physiotherapy in local centers to improve the general physical condition of older populations of cancer survivors, especially light-intensity activities [37,53]. Another potential application of these findings includes the implementation of decentralized physiotherapy pathways in ambulatory geriatric care settings, the integration of tele-rehabilitation strategies for rural populations, and the development of risk-adapted ERAS protocols tailored for frail oncology patients [34,54].

Several limitations of this study should be acknowledged. The first limitation of this study is the small size of the group, which limits the statistical power of the analyses and may reduce the ability to detect smaller changes in functional parameters and further generalization of our results. In addition, the absence of formal effect size estimation may limit the interpretation of the magnitude of observed changes despite statistical significance. Additionally, no a priori sample size calculation was performed due to the exploratory nature of the study and limited eligible population, which may have affected the statistical power. This reflects the clinical limitation in recruiting frail older adults for long-term follow-up. The study was conducted to assess the long-term effect of physiotherapy in patients aged ≥70 years with frailty, and a large group of patients, especially those living a long distance from the institution, did not agree to undergo the physical assessment a year after the surgery. Based on this finding, we assume that structured prehabilitation and its assessment in future trials should be realized in local ambulatory care units rather than University Hospitals. This should better enable the evaluation of tailored prehabilitation in older CRC populations. Second, the study was conducted at a large but single Colorectal Cancer Unit, which may have limited the generalizability of our findings. Third, the lack of randomization and the absence of a comparator group limit the ability to evaluate the exact causal relationships between prehabilitation and frailty progression. Additionally, selection bias and survivorship bias may have occurred and influenced the results, as only patients who survived surgery despite their age and remained sufficiently healthy to participate in the one-year follow-up assessment were included in the final analysis.

Despite these limitations, the study provides valuable long-term observational data on functional recovery in older adults with frailty undergoing CRC surgery, a cohort that remains underrepresented in longitudinal research. The results suggest that functional independence may remain relatively preserved during the first postoperative year in some patients, even in the presence of gradual physiological frailty progression.

Future studies should aim to address the limitations of the present investigation by incorporating larger multicenter cohorts and randomized controlled designs to better evaluate the potential impact of community-based multimodal structured prehabilitation programs integrated with long-term postoperative rehabilitation on long-term functional outcomes. Moreover, incorporating objective activity monitoring and patient-reported outcome measures may provide a more comprehensive understanding of functional recovery trajectories in frail CRC survivors aged ≥70 years. In addition, further research should explore the physiological mechanisms underlying the relationship between frailty, cardiopulmonary reserve, and postoperative recovery in older oncologic patients.

## 5. Conclusions

This study provides longitudinal observational data on long-term functional and physiological trajectories in older adults with frailty undergoing CRC surgery following a structured prehabilitation program.

Our main findings (only a subtle increase in 5-FI with stable CFS, improvement in postural function, and stable results for the 6MWT and the TUG test) suggest that functional performance may remain relatively stable over a 12-month observation period despite the slight increase in physiological frailty. These findings highlight a potential dissociation between physiological vulnerability and functional independence. These observations highlight the multimodal nature of frailty in this cohort.

However, due to the absence of a control group, the small sample size and the single-center design, the results should be interpreted as descriptive associations. Therefore, conclusions regarding the effectiveness of prehabilitation cannot be drawn.

Further large-scale, multicenter randomized controlled trials are required to determine the potential role of prehabilitation in influencing long-term functional outcomes, confirming the durability of observed results and optimizing multimodal prehabilitation strategies for frail older adults undergoing CRC surgery.

## Figures and Tables

**Table 1:** Summary of the clinicopathological features of the study group.

Characteristics	Study Group (*n* = 41)
Sex	
Male	27 (65.9)
Female	14 (34.1)
Age at diagnosis	75
Male	74.4
Female	76.2
BMI, kg/m^2^	27.8
18.5~24.9	10 (24.4)
25.0~29.9	18 (43.9)
≥30	13 (31.7)
History of diabetes	15 (36.6)
History of OSAS	1 (2.4)
History of AF	9 (22)
CAD	8 (19.5)
HTN	32 (78)
MI	3 (7.3)
Stroke	3 (7.3)
PCI	5 (12.2)
CABG	0
IBD	0
Other diseases	35 (85.4)
Other cancer	7 (17.1)
History of smoking	18 (43.9)
Pack-years	16.3
ASA	4 (9.8)
Steroids	7 (17.1)
Immunosuppression	1 (2.4)
Antiplatelets	6 (14.6)
Antithrombotics	8 (19.5)
Family history of CRC	8 (19.5)
Family history of other cancer	15 (36.6)
Hospitalization time	
Mean	6.6
Median	5
Rehospitalization	1 (2.4)
Tumor localization	
Colon	27 (65.9)
Rectum	14 (34.1)
Neoadjuvant therapy	12 (29.3)
Neoadjuvant RT	7 (50)
Neoadjuvant CHT	5 (12.2)
Neoadjuvant treatment toxicity	
Early	3 (7.3)
Late	0 (0)
Surgical approach	
Minimally invasive	7 (17.1)
Open	34 (82.9)
Operation time (min), median	131.3 (127)
Preoperative bowel preparation	
None	3 (7.3)
Fortrans	35 (85.4)
Enema	3 (7.3)
OAB	31 (75.6)
Clavien–Dindo scale	
Grade I	2 (4.9)
Grade II	17 (41.5)
Grade III	1 (2.4)
Grade IV	0 (0)
Non-surgical complications	16 (39)
Anastomotic leak	1 (2.4)
pT stage	
T1	5
T2	6
T3	25
T4	3
pN stage	
N0	28
N1–2	13
Lymph nodes	19.46 (16)
Adjuvant CHT	10 (24.4)
Early toxicity	2 (20)
Late toxicity	1 (10)

BMI—body mass index; OSAS—obstructive sleep apnea syndrome; AF—atrial fibrillation; CAD—coronary artery disease; HTN—hypertension; MI—myocardial infarction; PCI—percutaneous coronary intervention; CABG—coronary artery bypass grafting; IBD—inflammatory bowel disease; ASA—acetylsalicylic acid; CRC—colorectal cancer; RT—radiotherapy; CHT—chemotherapy; OAB—oral antibiotic; T—tumor; N—node.

**Table 2:** Summary of physical and physiological function assessment from baseline to 12-month follow-up.

Parameter	Before Prehabilitation	After One-Year Follow-Up	Mean Difference ± SD	95% CI	*p*-Value	Significance
Clinical Frailty Scale (CFS)	3.02	3.10	0.0732 ± 0.6079	−0.1187 to 0.2650	0.445	NS
5-item Frailty Index (5-FI)	0.28	0.31	0.0293 ± 0.0716	0.0067 to 0.0519	0.012	Significant
Physical activity score	1.97	1.63	−0.2667 ± 1.1123	−0.8780 to 0.3447	0.380	NS
Postural function score	0.67	0.93	0.4000 ± 0.9685	0.0384 to 0.7616	0.031	Significant
Chest circumference—inspiration	107.78	105.60	−1.08 ± 4.54 cm	−2.51 to 0.35	0.134	NS
Chest circumference—expiration	103.84	102.09	−0.86 ± 3.65 cm	−2.60 to 0.89	0.316	NS
Chest expansion delta (insp–exp)	4.02	3.51	−0.31 ± 1.61 cm	−0.96 to 0.32	0.319	NS

NS: not significant; SD: standard deviation; CI: confidence interval.

**Table 3:** Summary of functional capacity assessment from baseline to 12-month follow-up.

Parameter	Before Prehabilitation	After One-Year Follow-Up	Mean Difference ± SD	95% CI	*p*-Value	Significance
TUG test	12.36	10.42	−2.5231 ± 6.2959	−5.0661 to 0.0199	0.051	NS
6MWT distance	392.71	384.36	−2.8261 ± 92.8565	−42.9803 to 37.3281	0.885	NS
HR before 6MWT	76.92	73.03	−1.8333 ± 12	−6.9015 to 3.2348	0.462	NS
SBP before 6MWT	139.88	138.85	1.4167 ± 21.4231	−7.6295 to 10.4628	0.749	NS
DBP before 6MWT	79.48	73.87	−2.1667 ± 13.8208	−8.0027 to 3.6693	0.450	NS
MAP before 6MWT	99.61	95.53	−0.9722 ± 14.1004	−6.9263 to 4.9818	0.739	NS
SpO_2_ before 6MWT	98.16	96.87	−1.25 ± 2.02	−2.1059 to −0.3941	0.006	Significant
Borg scale before exercise	6.52	6.13	−0.4583 ± 1.2847	−1.0008 to 0.0841	0.094	NS
HR after 6MWT	88.58	87.87	3.6087 ± 21.2747	−5.5912 to 12.8086	0.425	NS
SBP after 6MWT	148.00	150.85	2 ± 14.7525	−4.3795 to 8.3795	0.523	NS
DBP after 6MWT	77.96	75.41	0.3478 ± 15.0925	−6.1786 to 6.8743	0.913	NS
MAP after 6MWT	101.31	100.56	0.8986 ± 13.1035	−4.7678 to 6.5649	0.745	NS
SpO_2_ after 6MWT	98.65	97.72	−0.9091 ± 0.9715	−1.3398 to −0.4784	<0.001	Significant
Borg scale after exercise	8.79	9.31	0.2609 ± 2.4903	−0.8160 to 1.3378	0.620	NS

NS: not significant; SD: standard deviation; CI: confidence interval; TUG—Timed Up and Go; 6MWT—six-minute walk test; HR—heart rate; SBP—systolic blood pressure; DBP—diastolic blood pressure; MAP—mean arterial pressure; SpO_2_: oxygen saturation.

## Data Availability

The data supporting the findings of this study are available from the corresponding author upon reasonable request.
